# Manifestations and pathological features of solitary thin-walled cavity lung cancer observed by CT and PET/CT imaging

**DOI:** 10.3892/ol.2014.2065

**Published:** 2014-04-15

**Authors:** YUANGANG QI, QING ZHANG, YONG HUANG, DAOQING WANG

**Affiliations:** 1 Department of Radiology, Affiliated Hospital of Shandong Academy of Medical Sciences, Jinan, Shandong 250032, P.R. China; 2 Department of Radiology, Shandong Cancer Hospital, Jinan, Shandong 250114, P.R. China

**Keywords:** lung cancer, body section radiography, pathology

## Abstract

The aim of the present study was to analyze and improve the understanding of computed tomography (CT) and positron emission tomography (PET)/CT imaging and the pathological features of solitary thin-walled cavity lung cancer. A total of 16 patients with pathologically confirmed solitary thin-walled cavity lung cancer were included in the present study. All of the patients received CT scans. Among these, two patients underwent an additional PET/CT examination. The CT and PET/CT images were analyzed and a cross-check analysis of the pathological results was conducted. In total, 16 cases of lesions demonstrated thin-walled cavities on the CT images. Among these cases, three presented with an uneven thickening of the cavity walls, 10 cases exhibited wall nodules and three cases presented with compartments in the cavity. The standard uptake value (SUV) of the cavity wall increased in two patients who underwent PET/CT examinations. The 16 cases of lesions were pathologically confirmed as adenocarcinomas. Light microscopy revealed that the tumor cells, which were observed in 12 cases of lesions, had diffused along the inner cavity wall and the tumor cells of four cases had invaded the bronchial wall. Images of the chest that demonstrated a single thin-walled cavity accompanied by uneven thickening of the cavity wall or wall nodules, in addition to an increase in the SUV and compartments in the cavity, indicated potential lung cancer. Valves formed as a result of bronchial wall damage may have led to the cavity.

## Introduction

Lung cancer is one of the most common types of malignant tumor (1,2) accounting for 22.7% of all cases of malignant tumor (3). The mortality rate of lung cancer in China has increased by 464.84% in the past 30 years (4). Furthermore, the incidence rate of lung cancer in China has exhibited the largest increase and poses the most severe threat compared with other types of cancer (5), therefore, an early and accurate diagnosis is required. Multi-slice spiral computed tomography (CT) is an important examination measure for the early screening of lung cancer and fully demonstrates the morphological characteristics of lung lesions (6,7). Typical types of lung cancer are easily diagnosed by imaging, biopsy and additional measures. However, there is a lack of knowledge concerning the features of atypical or rare types of lung cancer, thus, these types of cancer are subject to misdiagnosis, missed diagnosis and treatment delay. Among these types of cancer, solitary thin-walled cavity lung cancer is particularly rare (8–11). Doctors often lack the understanding of this type of cancer and, therefore, misdiagnose it. In the current study, 16 cases of thin-walled cavity lung cancer are included and summarized. CT and positron emission tomography (PET) manifestations, as well as the pathological features of these cases, were analyzed to determine their imaging characteristics with the aim of improving the diagnosis rate.

## Patients and methods

### General data

A total of 16 patients with pathologically confirmed thin-walled cavity lung cancer from the Shandong Cancer Hospital (Jinan, China) were enrolled in the present study between July 2008 and April 2012. These cases included 12 males and four females aged 34–69 years (mean age, 52 years). Among these, 11 cases did not manifest symptoms and were diagnosed only by physical examination. In addition, three cases suffered with a cough and chest-related distress, one case presented with hoarseness, tightness in the chest, pectoralgia and low-grade fever, and one case complained of right shoulder pain as well as a cough, which was accompanied by white phlegm. The present study was conducted in accordance with The Declaration of Helsinki and with approval from the Ethics Committee of Shandong Cancer Hospital. Written informed consent was obtained from all participants.

### CT examination

A 16-slice spiral CT scanner (Siemens Somatom Sensation Company, Erlangen, Germany) was used with a conventional-slice thickness of 5 mm. All of the lesions were reconstructed at a slice thickness of 1.5 mm and amplified. The patients were instructed to lie in a supine position, raise their arms, breathe in and hold their breath. Volume scanning was subsequently conducted. The scanning range began from the superior aperture of the thorax and ended at the posterior costophrenic angle of the left and right lungs. Abdominal scanning was conducted on certain patients and enhancement scanning was conducted on all patients. Nonionic contrast medium iohexol (Beijing BeiLu Pharmaceutical, Co., Ltd., Beijing, China; 300 mgI/ml, 80–100 ml) was used, and the injection rate ranged between 2.5 and 3.0 ml/sec. Scanning was conducted 30 and 90 sec after the contrast medium had been injected.

### ^18^F-fluorodeoxyglucose (FDG) PET/CT imaging method

A Discovery LS PET/CT instrument (GE Healthcare, Piscataway, NJ, USA) was used and ^18^F-FDG was generated by a PET tracer cyclotron (GE Healthcare) with a radiochemical purity of >95%. Following >4 h of fasting, the patients (in a tranquil state) were injected with the imaging agent ^18^F-FDG (259–444 MBq) via an intravenous T-shaped tube. The total body scan ranged from the mid-thigh to the top of the head. The PET and CT images were transferred to the eNTEGRA (GE Healthcare) workstation for the alignment fusion of the images.

### Imaging analysis

The CT images of the lesions were independently analyzed by two experienced imaging doctors to observe the cavity wall thickness, wall nodules, compartment locations and enhancement characteristics. In addition, the concentrations of ^18^F-FDG in the cavity wall nodules of the lesions were analyzed.

### Pathological detection

Biopsy samples were fixed in 10% neutral buffered formalin and dehydrated in a series of 50, 70 and 80% alcohols and distilled water. Following embedding in paraffin wax, sections were sectioned and stained with haematoxylin and eosin prior to examination under light microscopy (BX5IPF, Olympus Corporation, Tokyo, Japan).

## Results

### Findings from chest imaging

**Figure 1:**
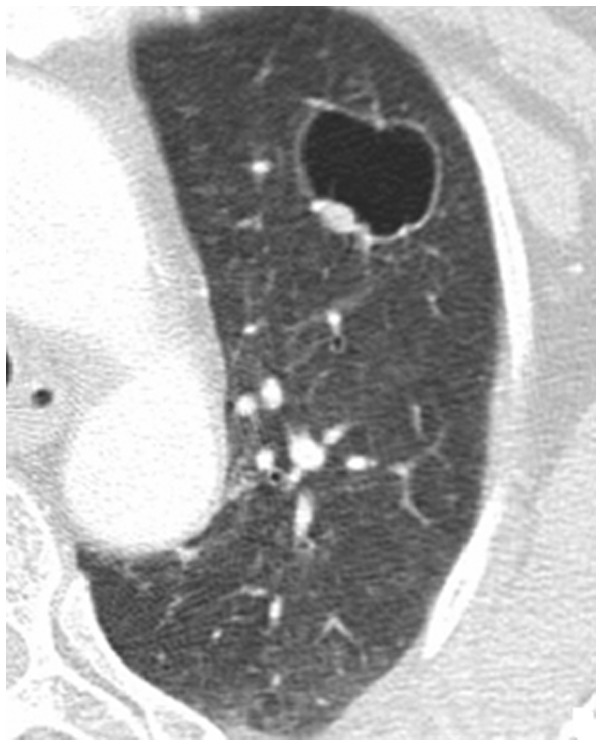
Male, 58 years old. The patient presented with uniform thickening of the cavity wall and wall nodules, however, there was no compartment in the cavity. The patient was diagnosed with moderately differentiated adenocarcinoma according to the pathological results.

**Figure 2:**
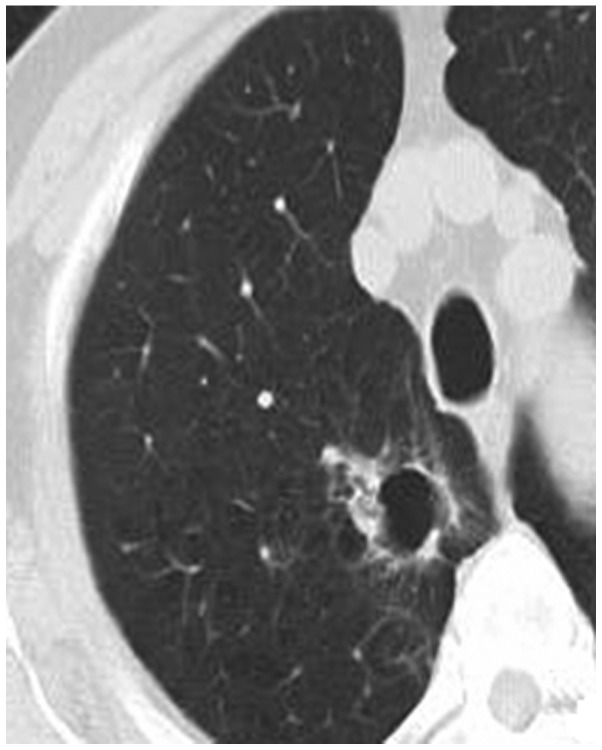
Male, 55 years old. The patient presented with uneven thickening of the cavity wall and wall nodules, however, there was no compartment in the cavity. The patient was diagnosed with highly differentiated adenocarcinoma accompanied by bronchial alveolar carcinoma according to the pathological results.

**Figure 3:**
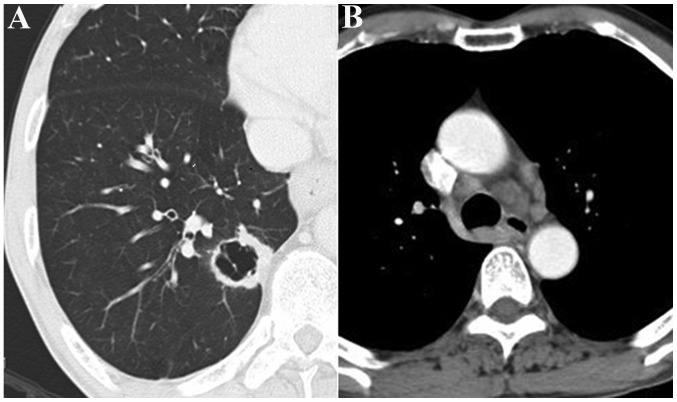
Female, 55 years old. The patient presented with (A) uneven thickening of the cavity wall and wall nodules, (B) multiple metastases of the mediastinal lymph node and partial lymph nodes fused into conglobation.

**Figure 4:**
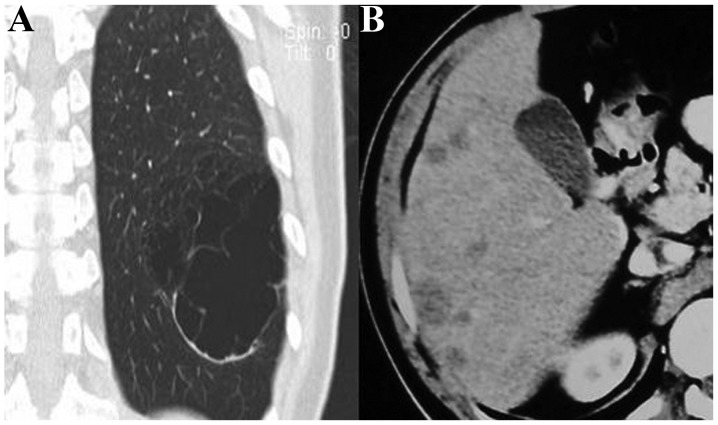
Male, 43 years old. The patient presented with (A) larger cavity, local thickening of the cavity wall and compartments in the cavity and (B) multiple metastatic tumors of the liver.

**Figure 5:**
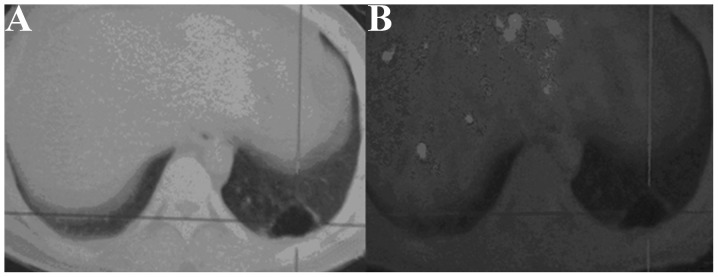
Male, 57 years old. The patient presented with (A) mild thickening of the cavity wall and (B) slight elevation of the standard uptake value (1.2).

**Figure 6:**
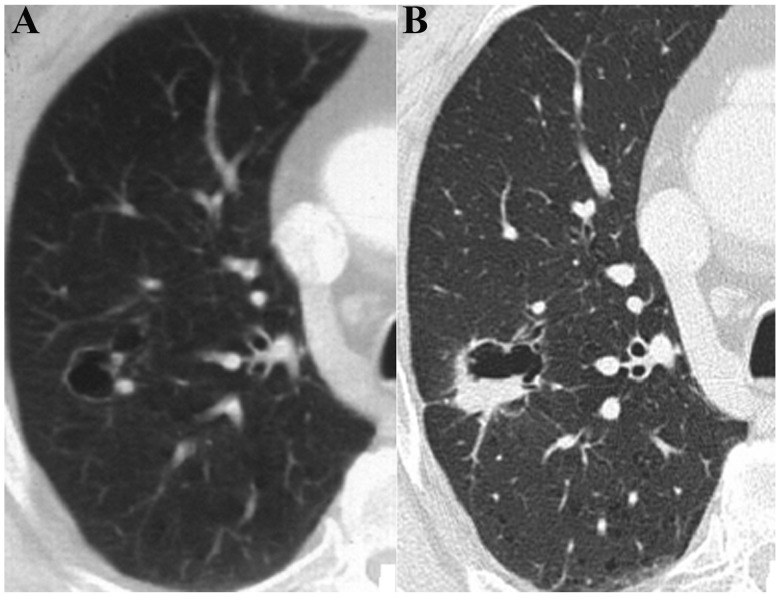
Male, 61 years old. The patient presented with (A) a cystic cavity in the posterior segment of the right lung upper lobe, no compartment, a slightly thickened wall and punctiform wall nodules. (B) Re-examination at month 18 demonstrated that the cavity was marginally enlarged, the wall was thickened, and the wall nodules were clearly enlarged and moderately enhanced. In addition, no compartments in the cavity were observed.

All of the thin-walled cavities of the 16 patients were located in the peripheral lung field and exhibited no signs of lobulation or spicules. Among these patients, three cases of lesions were located in the posterior segment of the right lung upper lobe, four were located in the posterior segment of the right lung lower lobe, two were located in the superior lobe apicoposterior segment of the left lung, three were located in the lower lobe basal segment of the left lung, three were located in the lower lobe posterior basal segment of the left lung and one in the lower lobe posterior basal segment of the left lung. The maximum cavity section was 6.5×5.2 cm and the minimum cavity section was 1.2×1.0 cm. The maximum size of the wall nodule was 1.0×0.8 cm and the cavity wall thickness was 0.3–1.2 mm. Two cases (12.5%) presented with a uniform thickening of the cavity wall and wall nodules (Fig. 1). Three cases (18.75%) presented with an uneven thickening of the cavity wall and wall nodules (Fig. 2). Three cases (18.75%) presented with an uneven thickening of the cavity wall (Fig. 3A) and multiple metastases of the mediastinal lymph node, and partial lymph nodes were fused into conglobation (Fig. 3B). One case (6.25%) presented with a large cavity, local thickening of the wall, compartments in the cavity (Fig. 4A) and multiple metastatic tumors of the liver (Fig. 4B). Three cases (18.75%) presented with compartments in the cavity and exhibited no signs of metastasis. Two cases (12.5%) presented with wall nodules on the cavity wall (Fig. 5A) and exhibited marginally increased standard uptake values (SUVs; Fig. 5B). One case (6.25%) presented with a cystic cavity in the posterior segment of the right lung upper lobe (Fig. 6A), no compartment, a wall thickness of ~1.0 mm and punctiform wall nodules. The cavity size was ~1.4×1.2 cm, no swollen lymph node was visible in the mediastinum, and the bilateral hilar and other organs demonstrated no signs of metastasis. Reexamination after 18 months demonstrated a marginally enlarged cavity (~1.6×1.5 cm), significantly enlarged and moderately enhanced wall nodules, a thickened wall (~2.0 mm) and no compartments or signs of metastasis in the cavity (Fig. 6B). For the other patient, a cystic cavity (~2.4×2.6 cm) was identified in the superior lobe apicoposterior segment of the left lung. The cavity wall presented local thickening and smooth edges and neither compartments nor signs of metastasis were identified in the cavity. Reexamination on the third and 12th month showed no variation in the lesions, and reexamination at 18 months demonstrated an enlarged cavity (2.8×2.2 cm) and wall nodules. Numerous swollen lymph nodes were observed in the mediastinum and the short diameter of the large lymph nodes was ~0.8 cm.

### Results of surgery and pathology

Six patients were not subjected to surgery due to mediastinal lymph node metastasis and liver metastasis. Therefore, biopsy samples were acquired by needle punctuation under CT guidance. For the 10 remaining cases, the affected lung was subjected to a partial lobectomy. The pathological results confirmed that the 16 patients were diagnosed with adenocarcinoma. Among these cases, four had moderately differentiated adenocarcinoma, five had highly differentiated adenocarcinoma accompanied by bronchial alveolar carcinoma, one had slightly differentiated adenocarcinoma accompanied by neuroendocrine carcinoma, two had mixed-type adenocarcinoma (bronchial alveolar carcinoma and alveolar-type adenocarcinoma) with visceral pleura involvement and four had highly differentiated adenocarcinoma.

### Pathological features

**Figure 7:**
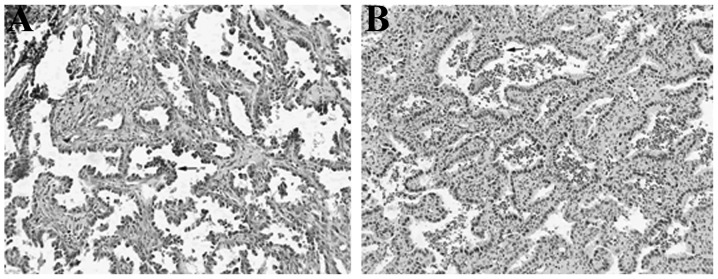
(A) Cancer cells directly diffused along the alveolar wall and pulmonary mesenchyme, and projected into the alveoli with a mammillary shape. The structures of the alveolar and bronchial walls were not damaged. (B) Cancer cells invaded the bronchial wall resulting in bronchial wall stenosis.

Light microscopy indicated that the cancer cells in 12 cases had directly diffused along the alveolar and bronchial walls and the pulmonary mesenchyme. Furthermore, the results demonstrated that the alveolar and bronchial walls were not damaged (Fig. 7A). In four cases, the cancer cells had invaded the bronchial wall, which resulted in bronchial wall stenosis (Fig. 7B). Necrotic tumor cells were not visible in any of the 16 patients.

## Discussion

Lung cancer is one of the most common types of malignant tumor and the progression of this type of cancer is associated with the pathological type. Solitary thin-walled cavity lung cancer is a unique and rare type of lung cancer, which is seldom reported. Therefore, this type of cancer is subject to misdiagnosis due to inadequate knowledge concerning its onset and progression. The improvement of diagnostic levels, particularly the invention of multi-slice spiral CT, has advanced our understanding of lung cancer. Cancer cells directly diffuse along the alveolar and bronchial walls, and the pulmonary mesenchyme. Bronchi stricture occurs and the structures of the alveolar and bronchial walls are not damaged (12,13). In other pathways, the cancer cells invade the bronchial wall, which results in bronchial wall stenosis (14). Therefore, the bronchi form a valve leading to excessive gas accumulation in the tumor that increasingly expands the cavities. Partial cavities subsequently break to form larger hollow cavities and tumors grow along the hollow cavity wall to form wall nodules or a cavity wall with an uneven thickness. In the present study, the cancer cells of six patients were observed to directly diffuse along the alveolar and bronchial walls, as well as the pulmonary mesenchyme. However, the alveolar and bronchial walls were not damaged. In 10 cases, the cancer cells invaded the bronchial wall, which resulted in bronchial wall stenosis. Necrotic tumor cells were not visible in any of the 16 patients.

Xue *et al* (15) reported that 18 cases of thin-walled cavity lung cancer were adenocarcinomas and in the present study, all 16 cases were adenocarcinomas. Among these cases, four had moderately differentiated adenocarcinoma, five had highly differentiated adenocarcinoma accompanied by bronchial alveolar carcinoma, one had slightly differentiated adenocarcinoma accompanied by neuroendocrine carcinoma, two had mixed-type adenocarcinoma (bronchial alveolar carcinoma and alveolar-type adenocarcinoma) with visceral pleura involvement and four had highly differentiated adenocarcinomas. These results are consistent with the study by Xue *et al* (15). Liu (16) also reported 25 cases of thin-walled cavity lung cancer. The pathological types included adenocarcinoma (21 cases), metastatic tumor (two cases), large-cell carcinoma (one case) and atypical hyperplasia (one case). Further studies are required to determine whether or not other types of cancer are present (for example, squamous cell and small-cell carcinoma).

In the present study, the mean age of the 16 cases was 52 years and the number of male patients compared with female patients was higher (12:4). The clinical manifestations of these patients varied with the lesion size. The tumors in the current study occurred in the periphery of the lungs. By contrast, Xue *et al* (15) described a different distribution pattern in 18 cases of cavity-type lung cancer. Given that the sample size of the current study is small, further studies are required to investigate whether the incidence rate is associated with gender and whether tumors primarily occur in the periphery of the lungs.

Few studies have examined thin-walled cavity lung cancer; its malignant signs are atypical, therefore, this type of lung cancer is easily missed during diagnosis. In the present study, 16 patients presented with cavities of varying sizes. The cavities contained gas, were located in the periphery of the affected lung and had no sign of lobulation or spicules. The partial cavities were of uneven wall thickness and their inner compartments were visible and became gradually enlarged. Specifically, the cavities of two patients were gradually enlarged and their wall nodules were pronounced. The partial-cavity patients exhibited thick cavity walls and presented with lymph node or organ metastasis. These CT observations are consistent with the malignant manifestations of thin-walled cavity lung cancer that have previously been observed (15) and are important signs for diagnosing this type of lung cancer. In the current study, the cavity wall of partial-cavity patients was thick and formed local nodules. Two patients exhibited thick cavity walls and PET demonstrated that the SUVs were 1.2 and 1.4 higher compared with normal values. Postoperative pathological results indicated a diagnosis of pulmonary adenocarcinoma. Given that the sample size of the present study was small, further investigations are required to determine whether or not these signs may be used as judgment standards for cystic lung cancer.

Solitary thin-walled cavity lung cancer is distinguishable from peripheral pulmonary cysts and thin-walled cavernous lung cancer. Pulmonary cyst walls are thin with uniform thickness, however, without wall nodules. Pulmonary cysts are not associated with compartments, lymphadenectasis or distant organ metastasis. In addition, long-term observations reveal no changes, and identification and diagnosis are simple to perform. CT signs of thin-walled cavernous lung cancer are widely reported and its malignant signs are clear, resulting in an easy diagnosis. For thin-walled cavernous lung cancer, the majority of pathological types belong to squamous carcinoma (17). The formation mechanism is characterized by extremely rapid tumor growth and central tumor-tissue necrosis, and necrotic components are formed by bronchial elimination. Imaging demonstrates that the cavernous wall is thicker than the cavity wall, the inner edges are rough, no compartments are found, and lobulation, spicules and pleural indentation are common.

In conclusion, thin-walled cavity lung cancer is rare with an incidence rate of 1.00–2.07% (17). Furthermore, there is a lack of knowledge regarding solitary thin-walled cavity lung cancer, therefore, it is easily misdiagnosed as a benign lesion, which delays treatment. Lung cancer may be indicated by the following clinical signs: Uneven thickening of the thin-walled cavity wall; wall nodule formation; the presence of compartments in the cavity; SUV value elevation; a thin-walled cavity accompanied by mediastinal lymph node or distant organ metastasis; or cavity enlargement under long-term observation. Further studies are required to determine whether certain signs, including an increase in the SUV of the cavity wall and enhancement of the wall nodule, may be used as judgment standards for solitary thin-walled cavity lung cancer.
